# Iberdomide enters multiple myeloma care: A new CELMoD with deep responses but uncertain long-term benefit

**DOI:** 10.1097/MS9.0000000000005701

**Published:** 2026-09-09

**Authors:** Basit Ali Shah, Javed Miandad, Ansar Said, Muhammad Qasim Safi, Nowshin Jamil Chaity, Marium Khan, Sharmin Rumana, Nazmun Naher Mouri

**Affiliations:** a Department of Internal Medicine, Saidu Group of Teaching Hospital, Swat, Pakistan; b Department of Internal Medicine, Lady Reading Hospital, Peshawar, Pakistan; c Department of Surgery, Khyber Teaching Hospital, Peshawar, Pakistan; d Department of Medicine, Ahsania Mission Cancer & General Hospital, Dhaka, Bangladesh; e Department of Anaesthesia, Dhaka Medical College, Dhaka, Bangladesh; f Department of Emergency and Accident, BRB Hospitals Ltd, Dhaka, Bangladesh

## Introduction

Multiple myeloma is a cancer of plasma cells in the bone marrow. Global Cancer Observatory (GLOBOCAN) estimates indicate approximately 188 000 new cases and 121 000 deaths worldwide in 2022, and the case count is expected to rise further as populations age^[^1^]^. Survival has improved a great deal over two decades, yet almost every patient relapses. After each relapse, there are fewer good options left, and the disease that comes back is harder to treat than it was the first time.

Much of that progress came from immunomodulatory drugs. Two of them, lenalidomide and pomalidomide, attach to a protein called cereblon, which sits inside the CRL4 ubiquitin ligase complex. Once the drug attaches, cereblon tags two proteins called Ikaros and Aiolos so that the cell breaks them down. The myeloma cell then loses signals it needs to stay alive^[^2^]^. These drugs stop working when the cell makes less cereblon or when other parts of the same pathway change. A patient who stops responding to lenalidomide usually gets little help from pomalidomide.

Iberdomide was designed for that problem. It binds cereblon about 20 times more tightly than lenalidomide and clears Ikaros and Aiolos more completely. It also keeps its effect in myeloma cells that resist both of the older drugs^[^3^]^. The gap in potency was wide enough that these compounds were placed in a class of their own, the cereblon E3 ligase modulators^[^4^]^.

## A new class of oral drug

Iberdomide reached patients first in a phase 1/2 study of iberdomide with dexamethasone. Those patients had late-line disease and had already received anti-CD38 antibodies as well as older drug classes. About one-quarter responded, and most adverse events were low blood counts^[^5^]^. A response rate near a quarter would count for little in early relapse. It counted for a great deal in a group with almost no standard options left.

Laboratory work then pointed to a better use of the drug. Iberdomide acted together with dexamethasone and daratumumab in cell studies, and the combined effect was greater than the effect of the single agents^[^6^]^. There is a reason for this. Once Ikaros and Aiolos are gone, T cells and natural killer cells work again. After daratumumab attaches to CD38, natural killer cells do much of the killing that follows. So a tablet that puts immune cells back to work fits well with an antibody. Figure 1 shows the steps inside and around the myeloma cell. Table 1 lists iberdomide next to the other drugs that act on the same target.

**Figure 1.**
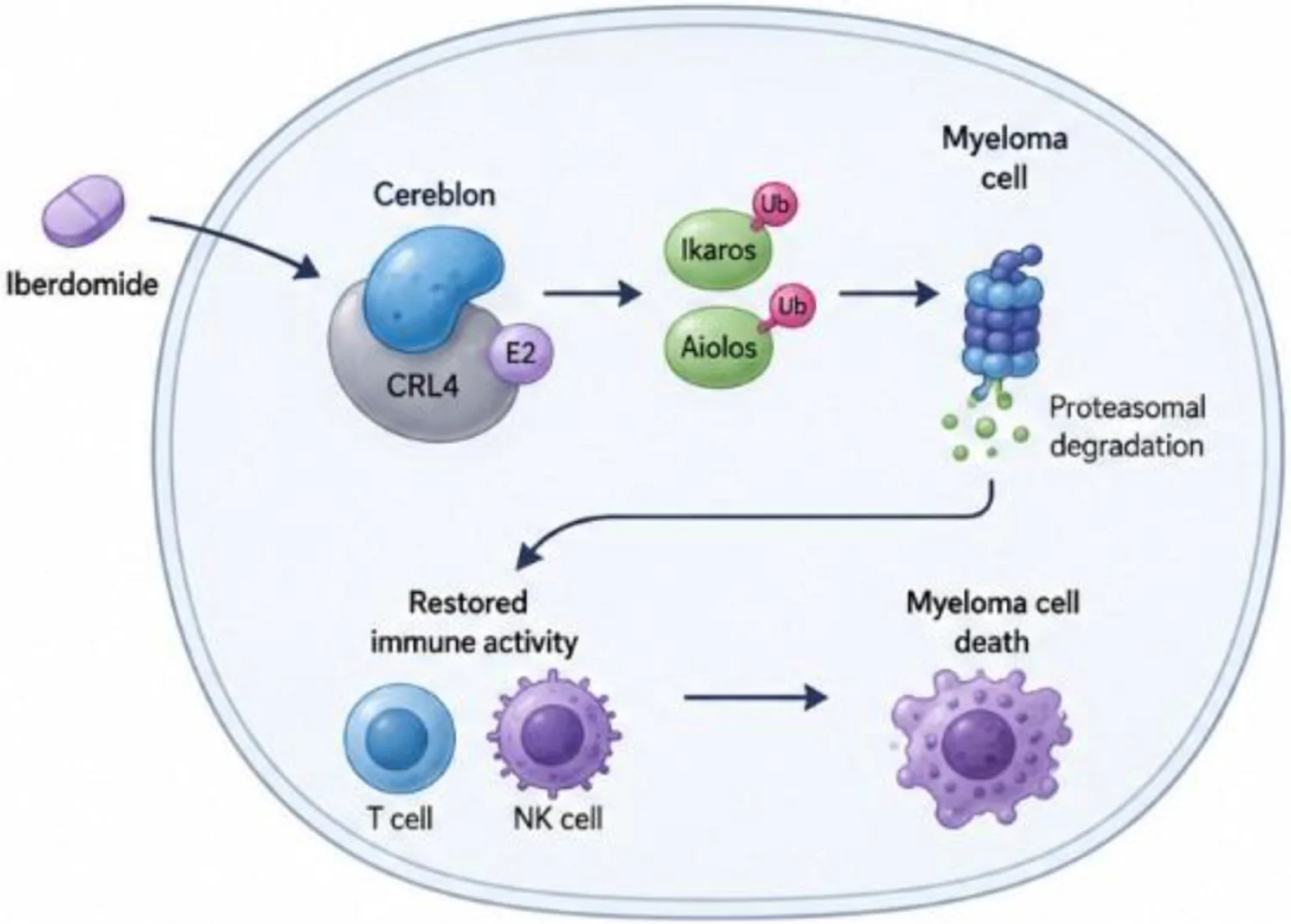
Action of iberdomide through cereblon.

**Table 1:** Drugs that act through cereblon in multiple myeloma.

Agent	Class	Route	Cereblon binding against lenalidomide	Status in myeloma (August 2026)
Thalidomide	IMiD	Oral	Weaker	Approved with dexamethasone
Lenalidomide	IMiD	Oral	Reference	Approved across several settings
Pomalidomide	IMiD	Oral	Similar	Approved after lenalidomide and a proteasome inhibitor
Iberdomide	CELMoD	Oral	About 20 times stronger	Approved with daratumumab and dexamethasone after one prior line
Mezigdomide	CELMoD	Oral	Stronger	Not approved, under phase 3 study

One point about the trial design is worth mentioning. The dose taken forward was 1 mg once daily, which is lower than the 1.6 mg dose used with dexamethasone alone in late-line disease^[^5^]^. Regulators now want companies to compare doses before they run a confirmatory trial rather than take the highest tolerated dose straight into it, and stage 1 of EXCALIBER-RRMM did that. The dose matters here because iberdomide lowers neutrophil counts, and it is given with an antibody that also makes infection more likely.

## Evidence behind the approval

EXCALIBER-RRMM tested the combination in early relapse. The trial used a seamless design in two stages. Stage 1 compared three doses of iberdomide with daratumumab and dexamethasone against daratumumab, bortezomib, and dexamethasone, and stage 2 carried the selected 1 mg dose forward against the same control^[^6^]^. Enrollment reached 939 patients across 259 sites, with all patients having one or two prior lines of therapy^[^7^]^. The control regimen was a fair choice because daratumumab with bortezomib and dexamethasone became a standard after the CASTOR trial reported a clear gain in progression-free survival over bortezomib with dexamethasone alone^[^8^]^.

The result behind the approval comes from the first 420 patients randomly assigned: 207 to the iberdomide triplet and 213 to the control. A complete response with no detectable residual disease at any time occurred in 41 percent of the iberdomide group, with a 95% confidence interval of 34–48%, and in 21% of the control group, with an interval of 15–27%. The difference reached a *P*-value below 0.0001^[^9^]^. Figure 2a presents both rates, and Figure 2b sets out the trial structure.

**Figure 2.**
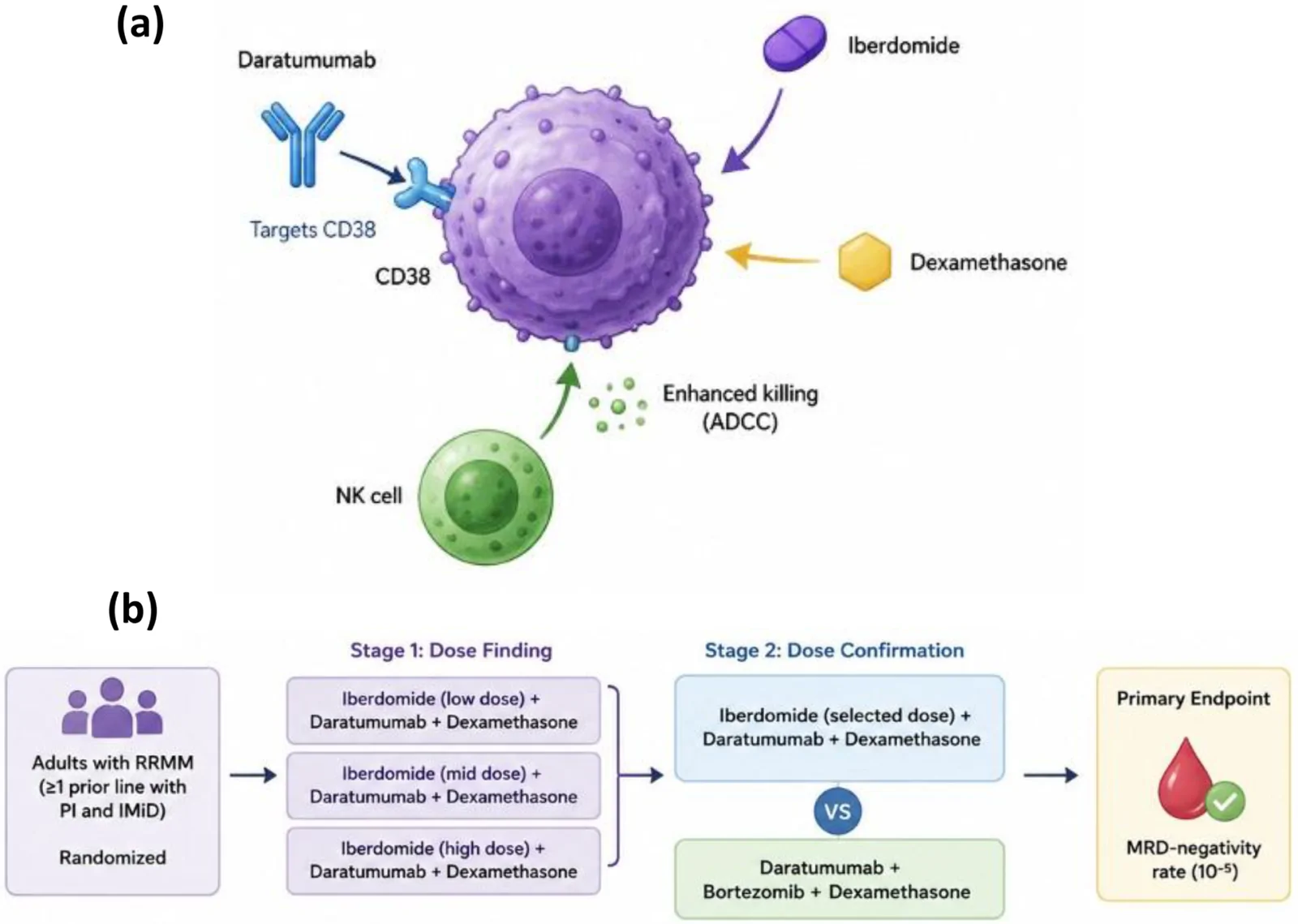
(a) Rates of deep response in EXCALIBER-RRMM. (b) Trial design and the reported endpoint.

The size of that difference deserves plain description. The two rates differ by 20 percentage points, and the iberdomide arm reached about twice the proportion seen in the control arm. One feature of the endpoint should temper the interpretation. A negative result counted at any point during the study, and the length of treatment was not equal between the arms, since bortezomib is stopped after eight cycles, whereas iberdomide continues until progression. A patient who stays on treatment longer has more opportunity for a deep response to appear. A gap this wide is too large for that to explain on its own, but a fair interpretation of the data should still note it.

The review moved fast. Iberdomide already held breakthrough therapy and orphan drug status. The application received priority review, and the agency assessed it under Project Orbis, together with Swissmedic in Switzerland^[^9^]^. Sagar Lonial of the Winship Cancer Institute, who worked on the trial, said the decision brought a new class of therapy to patients with relapsed or refractory disease^[^10^]^. That is a fair thing to say about how the drug works. It is not yet a fair thing to say about how long patients live.

## Safety and everyday use

The label carries a boxed warning for two harms. One is damage to the fetus, and the other is serious clotting in veins or arteries^[^9^]^. The first harm is well known from thalidomide and the drugs that came after it, and it is the reason patients can only get the drug through a restricted safety program. The second harm matters because myeloma already raises the risk of clots, so most patients will need aspirin or an anticoagulant along with the triplet. Neutrophil counts drop often, and infections were among the most common side effects. The label lists pneumonia and upper respiratory infection, and it also lists low immunoglobulin levels^[^9^]^. Doctors who use daratumumab already know this pattern, because anti-CD38 treatment cuts normal antibody production too. The label also warns about second primary cancers, and the warning fits what doctors have seen with this drug family for years. Table 3 summarizes these points.

There is a gain on the other side. The new regimen does not include bortezomib, so patients avoid the drug that causes most of the nerve damage in this setting. Iberdomide is a capsule taken once a day for the first 21 days of a 28-day cycle. From cycle 7, the antibody is given as a monthly injection. Table 2 sets the two regimens next to each other.

**Table 2:** The two regimens compared in EXCALIBER-RRMM.

Feature	Iberdomide, daratumumab, and dexamethasone	Daratumumab, bortezomib, and dexamethasone
Partner drug	Iberdomide 1 mg by mouth, days 1–21 of a 28-day cycle	Bortezomib 1.3 mg per square meter under the skin, days 1, 4, 8, and 11 of a 21-day cycle
Antibody	Daratumumab and hyaluronidase 1800 mg under the skin, weekly then less often	Daratumumab and hyaluronidase 1800 mg under the skin, weekly then less often
Steroid	Dexamethasone 20 mg or 40 mg by mouth once a week	Dexamethasone 20 mg by mouth on 8 days of each 21-day cycle
Planned duration	Until disease progression or unacceptable toxicity	Bortezomib for eight cycles, then daratumumab alone
Main toxicity concern	Low neutrophils, infection, and clot risk	Peripheral neuropathy and low platelets
Deep response rate	41%	21%

**Table 3:** Label warnings and common adverse reactions with the iberdomide triplet.

Category	Detail
Boxed warning	Harm to the fetus and serious venous or arterial thromboembolism
Other warnings	Neutropenia, infection, and second primary cancer
Risk control	Restricted distribution through a dedicated safety program
Common adverse reactions	Upper respiratory infection, fatigue, muscle and bone pain, pneumonia, diarrhea, rash, poor sleep, and low immunoglobulin levels
Grade 3 or 4 laboratory changes	Reduced neutrophils, white cells, and lymphocytes

A hematology service will not find the monitoring difficult. Blood counts need to be checked at each cycle. A short delay in dosing, or some growth-factor support, will cover most episodes of low neutrophil counts. Immunoglobulin replacement has a role in patients with recurrent infections, and vaccination is best arranged before the start of treatment when time allows. Little of this differs from the care already provided with an anti-CD38 antibody, which is one reason the regimen should transfer easily into general practice.

## Minimal residual disease as the basis for approval

This approval is as notable for its endpoint as it is for its drug. Minimal residual disease refers to myeloma cells that remain in the marrow below the level detected by routine tests. The International Myeloma Working Group set criteria in 2016 and defined negativity as one tumor cell in 100 000 or fewer, measured by flow cytometry or sequencing, in a patient who already meets the definition of complete response^[^11^]^.

The prognostic value of that measure is well established. The EVIDENCE meta-analysis pooled patient-level data from randomized trials and found a close link between negative status and later outcomes^[^12^]^. Work at the trial level pointed in the same direction, since treatment effects on residual disease tracked treatment effects on progression-free survival^[^13^]^. An advisory committee of the agency voted without dissent in April 2024 that the endpoint was reasonably likely to predict clinical benefit and draft guidance for industry followed^[^14^]^.

Therefore, the endpoint has support. The caution lies elsewhere. A measure taken early tells us that a regimen clears disease more deeply, not that patients live longer or live better. In cancer medicine, many drugs received accelerated approval and then went years without proof, and a few lost the approval when the confirmatory trial failed^[^15^]^. EXCALIBER-RRMM will report progression-free survival and overall survival once follow-up is longer, and iberdomide will be judged on those numbers. Until then, the fair reading is that the drug clears disease more deeply than a familiar comparator. That is a reason to use it in the right patient. It is not a reason to rewrite every treatment plan.

## Use in practice

Think of a patient in a first or second relapse who has had a proteasome inhibitor and lenalidomide. For that patient, the new triplet retains the anti-CD38 antibody and adds an oral tablet to it. It is a good fit for someone with severe neuropathy and also for someone who has to travel a long way for treatment. It is not a fit for a patient whose disease no longer responds to daratumumab or to bortezomib, because the trial excluded both groups^[^7^]^. Patients who had previously taken iberdomide were also excluded, so the label applies to people who have never received a drug of this class.

The harder question is where the drug belongs in the order of treatments. Myeloma care now includes bispecific antibodies and CAR T-cell products for later lines, and both work through T cells. A drug that brings T cells back to work might prepare the ground for those treatments, or it might wear the same cells out. Nobody knows yet, and the answer will decide where this class sits in a treatment plan. Cost and access will also decide how often the drug is used. A tablet that a patient takes until the disease gets worse runs up a long bill for health systems and patients.

Patients need a plain account of what the approval means. A deeper remission is worth having, and it may lead to longer control of the disease, but no one has shown that yet. Doctors should say so before they write the prescription, and they should also say what the drug will cost over years of use.

## Conclusion

Iberdomide introduces a new drug class into routine myeloma care. It is an oral tablet and works with an antibody that many patients already receive. Twice as many patients achieved a deep response as with an accepted comparator, and the result is reliable. A regimen without bortezomib will also suit patients who are at risk of nerve damage. Proof of benefit still awaits survival data from a trial that has not finished. For now, the safe course is careful use in patients who match the trial population.

## Data Availability

Data sharing does not apply to this article as no new data were created or analyzed in this study.

## Research registration unique identifying number (UIN)

Not applicable (This is a commentary article and does not report the results of a clinical trial).
